# Accessing scientific data through knowledge graphs with *Ontop*

**DOI:** 10.1016/j.patter.2021.100346

**Published:** 2021-10-08

**Authors:** Diego Calvanese, Davide Lanti, Tarcisio Mendes De Farias, Alessandro Mosca, Guohui Xiao

**Affiliations:** 1 Faculty of Computer Science, Free University of Bozen-Bolzano, 39100 Bolzano, Italy; 2 Department of Computing Science, Umeå University, 901 87 Umeå, Sweden; 3 Ontopic S.R.L., 39100 Bolzano, Italy; 4 SIB Swiss Institute of Bioinformatics, 1015 Lausanne, Switzerland; 5 Department of Ecology and Evolution, University of Lausanne, 1015 Lausanne, Switzerland

**Keywords:** ontology-based data access, virtual knowledge graphs, data integration, ontology language, biomedical data

## Abstract

In this tutorial, we learn how to set up and exploit the virtual knowledge graph (VKG) approach to access data stored in relational legacy systems and to enrich such data with domain knowledge coming from different heterogeneous (biomedical) resources. The VKG approach is based on an ontology that describes a domain of interest in terms of a vocabulary familiar to the user and exposes a high-level conceptual view of the data. Users can access the data by exploiting the conceptual view, and in this way they do not need to be aware of low-level storage details. They can easily integrate ontologies coming from different sources and can obtain richer answers thanks to the interaction between data and domain knowledge.

## The bigger picture

Knowledge graphs (KGs) have recently gained attention due to their flexible data model, which reduces the effort needed for integration across different, possibly heterogeneous, data sources. In this tutorial, we learn how to access scientific data stored in a relational database through the virtual knowledge graph (VKG) approach. In such an approach, the data are exposed as a KG and enriched with semantic information coming from a domain *ontology*. The KG is “virtual” in the sense that the data are not replicated but stay within the data sources and are accessed at query time.

We demonstrate the approach over scientific data coming from the biomedical domain and using the open-source VKG system *Ontop*. Since legacy data are exposed as a KG, users can access the data by means of a more convenient vocabulary provided by the domain ontology, benefit from automated reasoning capabilities, and do not need to focus on how the data are actually stored. Furthermore, the virtual approach allows for the use of KGs even in those contexts where the user does not own the data nor is granted the rights to make a copy of them.

By relying on existing federation tools, the approach described here for accessing scientific data can also be used to integrate multiple, heterogeneous, and possibly semi-structured and unstructured data sources.

## Introduction and motivation

Large-scale molecular biology experiments, the adoption of computational tools and algorithms to study biological pathway networks,^1^ the effective analysis of genome sequences from various model organisms, and, more generally, the advent of a systems approach for the analysis and modeling of complex biological systems,^2^^,^^3^ all require advanced data management technologies that ease the access to and the integration of massive amounts of information coming from different, usually heterogeneous, data sources.

Knowledge graphs (KGs) gained popularity recently^4^ as a general mechanism to represent data that are not constrained to a rigid schema, and to enrich such data with domain semantics. The absence of a rigid schema, and the elementary yet flexible abstraction provided by “subject-predicate-object” triples at the basis of KGs, allow on the one hand for the evolution of data sources in all those situations where the “schema” of the data cannot be determined in advance, and on the other hand for the integration and interoperability of heterogeneous data sources and among different scientific data platforms.

The semantic information in a KG is provided by an *ontology*, which is a structured formal representation of the concepts that are relevant in a domain of interest and of the relationships between them. The purpose of the ontology is 2-fold. On the one hand, it defines a vocabulary of terms to denote *classes* and *properties* that are familiar to the user. On the other hand, it extends the data with background knowledge, such as sub-class and sub-property axioms, axioms establishing which classes constitute the domain and range of properties, and axioms expressing the disjointness between classes or properties.

The data in a KG consists of a set of *data assertions* that use the vocabulary of classes and properties provided in the ontology. Data assertions are often obtained by mapping the data stored in various data sources to the terms of the ontology vocabulary. Intuitively, a *mapping* can be thought of as a collection of queries that are used to construct the data assertions of the ontology by retrieving the necessary data from the sources.

The data sources are typically legacy systems and might come in different forms, such as relational databases (DBs), or as files in various formats (such as CSV, XML, JSON, or proprietary formats). For the purpose of this tutorial, we assume to deal with a single relational data source. To deal with multiple heterogeneous data sources one can resort to a data federation tool, such as Denodo,^5^ Dremio,^6^ or Teiid,^7^ which expose such sources as if they were part of a single relational DB.

KGs, through an explicit and non-ambiguous representation of the semantics of the data, promote:

• interoperability among different scientific *data platforms* (e.g., GDC^8^ and ELIXIR,^9^ *resources* (e.g., UBERON^10^ and CHEBI,^11^ and *data models* (e.g., SBML^12^ and BioPAX^13^);
• scientific reproducibility and replicability of experimental studies;
• knowledge discovery and data mining practices by exposing a conceptually sound view over a multiplicity of distinct and possibly non-interoperable data sources, therefore reducing the negative impact of inputting nonsensical or inconsistent data into statistical models and learning algorithms;
• enrichment of the information originally present in the data sources through the application of reasoning techniques that combine domain knowledge and data assertions.

In a virtual knowledge graph (VKG),^14^^,^^15^ the data assertions are not materialized in a separate data store, but their presence in the KG is only virtual. Systems operating on VKGs are able to retrieve the data directly from the data sources only when it is required for a particular user query. In fact, query processing is delegated to the data sources. This is achieved by unfolding the mappings, thus translating user queries into queries over the data sources, while taking into account also the ontology background knowledge through a so-called query-rewriting step. The advantage of VKGs is that information is always fresh and up-to-date with the data sources.

Despite the advantages of the virtual approach, it is sometimes convenient to actually materialize the data assertions. In such a case, we talk about materialized knowledge graphs (MKGs). The main advantage of MKGs over VKGs is that usually a better performance in query answering can be achieved, especially in those situations where mappings are very complex and thus the unfolding of the virtual approach would give rise to complex queries over the data sources. This comes at the cost of maintaining a potentially very large MKG.

**Figure 1:**
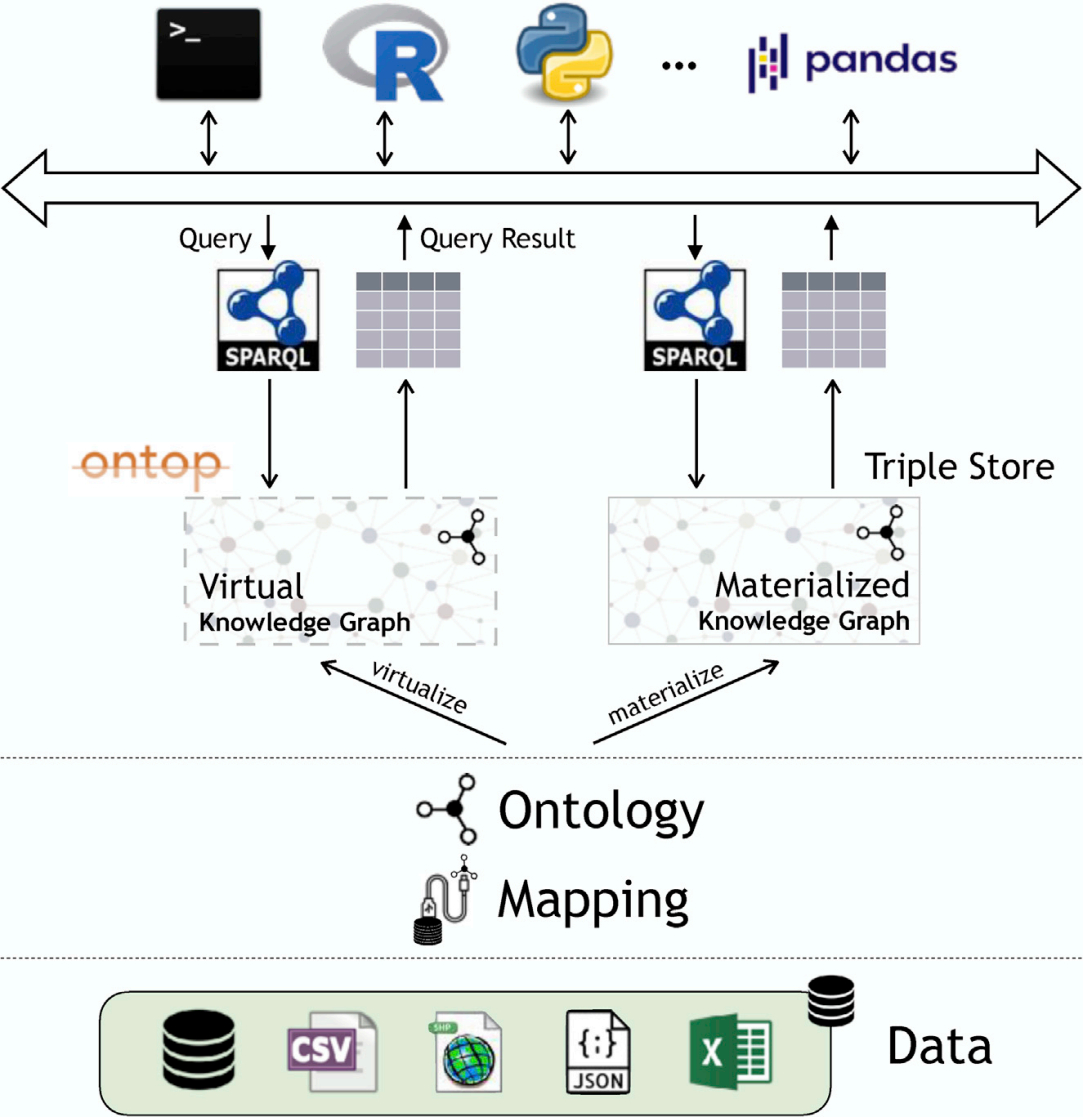
KG conceptual framework

Figure 1 shows the conceptual framework of KGs, in both the materialized and virtual flavors. The elements of the KG framework are expressed in formal languages standardized by the World Wide Web Consortium (W3C), specifically: the KG in RDF,^16^ the ontology in OWL 2 QL,^17^ the mapping in R2RML,^18^ and the query in SPARQL.^19^

In this tutorial we make use of the VKG system *Ontop*^20^^,^^21^ to set up a KG in the biomedical domain, specifically in the area of cancer research.

We observe that *Ontop* has been conceived as a VKG system, but it offers also functionalities for materializing a KG from a VKG specification consisting of an ontology, a relational data source, and a mapping between the two.

All the material used in this tutorial, as well as full details for its usage, are available in an online GitHub repository.^22^

### Related work on VKG systems

Among the open-source VKG systems, *Ontop* is one of the most popular (with over 30K+ downloads in the past 5 years, according to Sourceforge). *Ontop* is a state-of-the-art VKG system initially developed by the Research Center for Knowledge and Data (KRDB) at the Free University of Bozen-Bolzano, and currently maintained as a community effort, involving both academic institutions and companies (most notably, Birkbeck University of London and Ontopic S.R.L.). The system has been adopted in many academic projects (most notably, the two European projects FP7 Optique^23^ and H2020 INODE^24^), and it also has a number of commercial deployments, such as the *UNiCS* open data platform by SIRIS Academic^25^ (Spain) and the Open Data Hub Virtual Knowledge Graph project for publishing tourism data of South Tyrol (Italy). All the authors of this tutorial have a profound expertise in the system, some being the maintainers since its inception.

An overview of popular commercial and non-commercial VKG systems, as well as a comparison between *Ontop* and other systems, goes beyond the scope this work. For such aspects, we refer the interested reader to the vast scientific literature.^15^^,^^26^, ^27^, ^28^, ^29^

## The EasyBgee dataset

Gene expression is a key process to understand the relations between genes and their function. It indicates or mediates the gene implication in functions, disease development, and organism, species or gene diversity. In this context, the Bgee DB^30^ is a public relational DB that consolidates and curates heterogeneous gene expression data sources.^31^

**Figure 2:**
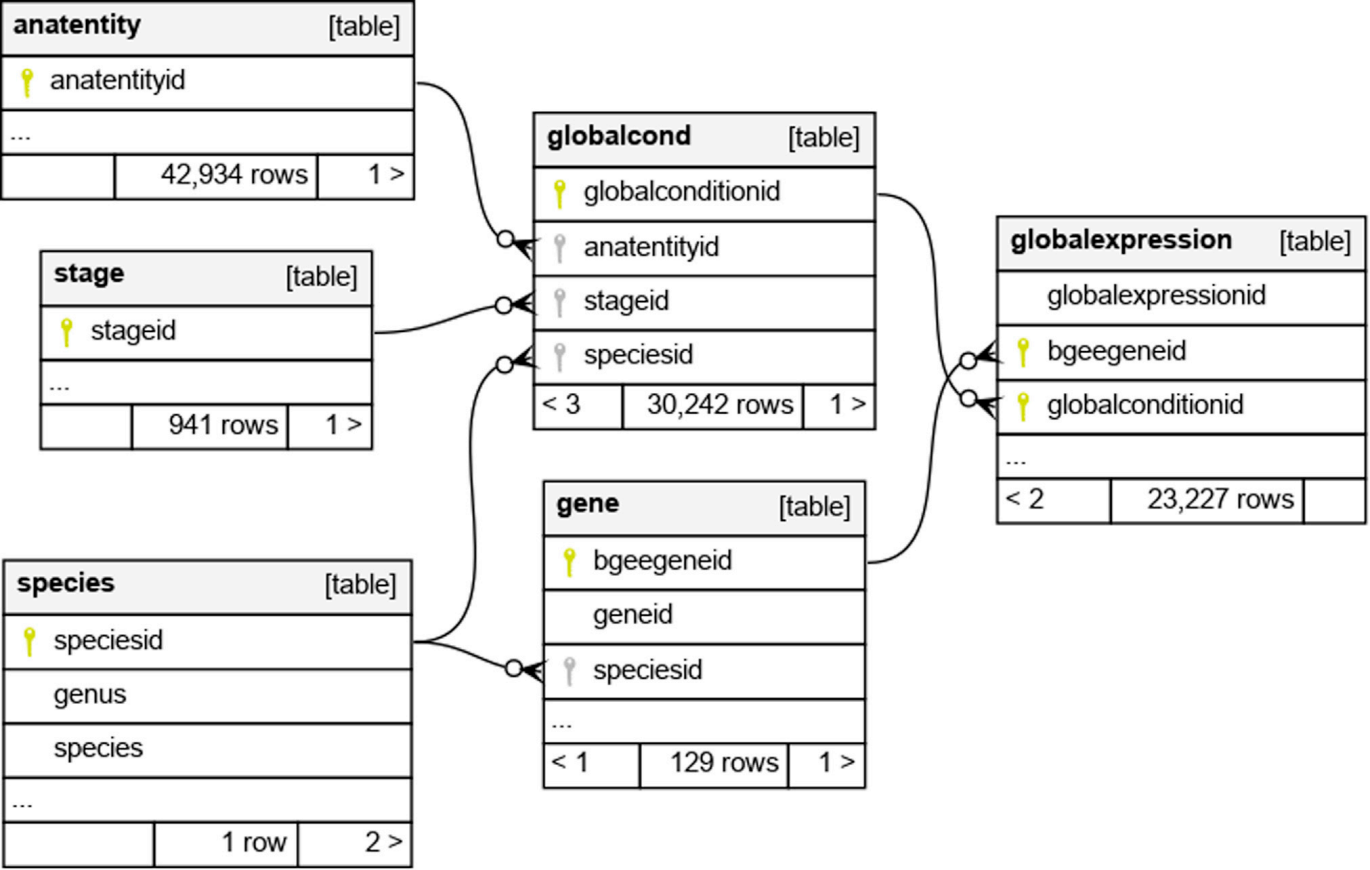
EasyBgee data schema (portion)

Figure 2 illustrates the data schema of the EasyBgee DB (which is available as a MySQL dump^32^^,^^33^), a simplified version of the Bgee DB. Currently, EasyBgee 14.2 contains gene expression data of 29 species. In this tutorial, we consider a subset of EasyBgee to explain and demonstrate the main principles of the KG approach to data integration and data access. This subset has exactly the same data schema as the entire EasyBgee DB, however, with considerably fewer data. It solely includes data related to 129 genes out of 17,559 in the fruit fly species (i.e., *Drosophila melanogaster*). As a result, this subset corresponds to less than 8 MB of data when serialized in a MySQL or PostgreSQL dump text format. The DB subset dump is available for download in the GitHub repository of the tutorial.

As shown in Figure 2, EasyBgee consists of 6 tables, 29 columns, and 6 foreign key constraints (represented with arrows). We provide a brief description of the tables:

• the anatentity table contains data about anatomic entities, such as organs (e.g., “brain”);
• the stage table describes developmental stages related to several species (e.g., the “egg stage”);
• the gene table contains the gene names and descriptions from different species;
• the species table contains information about animal species, such as their scientific and common names;
• the globalcond table contains the experimental conditions of a gene expression analysis, such as the species, its developmental stage, and the anatomical entity considered in the analysis;
• the globalexpression table contains the gene expression patterns by relating with a score a gene to an experimental condition where the gene is expressed or absent.

## Setting up a VKG

In this section we discuss how to set up an instance of a VKG system by means of a concrete use case coming from the domain of bioinformatics.

### Gene expression ontology

A crucial step in the deployment of a VKG system consists in the design or re-use of an ontology that suitably represents the implicit semantics of the underlying data. For the sake of the present tutorial, in the gene expression domain we highlight the gene expression ontology (GenEx),^34^ which is specifically designed to structure gene expression data from DBs, such as EasyBgee. In addition to new terms defined in the ontology itself, GenEx imports terms from different vocabularies, such as relation ontology (RO).^35^ As an example, GenEx imports from RO the “expressed in” property (actually identified by obo:RO_0002206) and its inverse property “expresses” (identified by obo:RO_0002292). Examples of data assertions using these properties are: “the insulin gene is *expressed in* the body of pancreas,” and its inverse statement, “the body of pancreas *expresses* the insulin gene.” Moreover, GenEx specializes the RO “expressed in" property by defining genex:isExpressedIn as its sub-property with a specific domain and range (see also the bottom part of Figure 4). The domain and range of genex:isExpressedIn include orth:Gene and genex:AnatomicalEntity, respectively. Therefore, we can assert that a gene is expressed in an anatomical entity (e.g., the body of the pancreas) using the genex:isExpressedIn property and automatically infer a more general statement about its RO super-property. Similar definitions in the ontology contribute to enhance interoperability because different ontology terms (RO and GenEx specific) can be interchangeably used to retrieve gene expression calls. Notice that all terms are prefixed with labels that indicate the ontology they were originally defined in and that allow for compact identifiers (called URIs, in RDF terminology). The prefixes used in the present article, such as genex: and orth:, are defined in Table 1.

**Table 1:** The namespace prefixes used in this tutorial

Prefix	Namespace IRI
rdf:	http://www.w3.org/1999/02/22-rdf-syntax-ns#
rdfs:	http://www.w3.org/2000/01/rdf-schema#
owl:	http://www.w3.org/2002/07/owl#
xsd:	http://www.w3.org/2001/XMLSchema#
orth:	http://purl.org/net/orth#
up:	http://purl.uniprot.org/core/
obo:	http://purl.obolibrary.org/obo/
dcterms:	http://purl.org/dc/terms/
genex:	http://purl.org/genex#
oma:	http://omabrowser.org/ontology/oma#

IRI, internationalized resource identifier.

Furthermore, GenEx reuses other ontology terms not only as part of its terminology but also as part of the data assertions by acting as a controlled vocabulary. For example, in GenEx, the Uber-anatomy (UBERON) ontology classes are considered as instances of the classes genex:AnatomicalEntity or efo:EFO_0000399 (i.e., “developmental stage”) by punning.^36^ The classes of the species-specific developmental stage ontologies^37^ are also considered as instances of the efo:EFO_0000399 class, which has been imported from the experimental factor ontology (EFO).^38^ Therefore, these controlled vocabulary terms are assigned as specific values of the genex:isExpressedIn property. As a result, we enhance semantic and data interoperability with other data sources that also adhere to the same controlled vocabularies. For example, while re-using the term obo:UBERON_0000955 (labeled as “brain”) in our data assertions, we know that we are referring without any ambiguity to the very same organ as the one defined in the UBERON ontology. Notice also that, by applying the above strategy, we enable the enrichment of the information coming from the original data sources with further assertions coming from the ontology specification. For example, this enrichment allow us to retrieve the UBERON data assertions, such as cross-references (i.e., similar terms), related synonyms, and “part of” assertions (e.g., “the brain is *part of* the central nervous system”) that are not available in the original data source (e.g., EasyBgee). For further details about GenEx, please consult its documentation.^34^

### Mapping EasyBgee to GenEx

The second main step in deploying a VKG system consists in the specification of its mapping. If we consider the EasyBgee data source, we can observe that for each of its tables there is at least one corresponding class in GenEx. Now, to populate GenEx using a system, such as *Ontop*, we need to define suitable mapping assertions from the tables we are interested in to their corresponding classes and properties in the ontology. Each of these mapping assertions consists of three components:

1. The *mapping identifier*, which uniquely identifies the mapping assertion.
2. The *source* part, which is an ordinary SQL query expressed over the (relational) data source.
3. The *target* part, consisting of a set of RDF triple patterns that make use of the answer variables of the SQL query in the source part. Such answer variables are written by enclosing them in curly brackets ‘{’ and ‘}’.

obo:{anatEntityIdSPARQL} a

genex:AnatomicalEntity .

obo:{anatEntityIdSPARQL} dcterms:description

{anatEntityDescription} .

obo:{anatEntityIdSPARQL} rdfs:label

{anatEntityName} .

The meaning of such a mapping assertion is that it constructs a portion of the KG as specified in the target part, by retrieving the required data from the data source through the SQL query in the source part. More specifically, for each answer tuple $\vec{t}$ returned by such SQL query, the triples in the target part are asserted to hold in the KG constructed by the mapping. These triples are obtained by substituting in the triple pattern each SQL answer variable (enclosed in ‘{’ and ‘}’) with the corresponding value in the answer tuple $\vec{t}$. We remark that, when the KG is kept *virtual*, the triples are actually not constructed, but SPARQL queries are answered over the VKG (as if they were evaluated over such triples) by unfolding them to (SQL) queries over the data source.

Let us also notice that the mappings that are introduced in this tutorial are specified in the *Ontop* native mapping language, which is easier to learn and use. However, *Ontop* allows users to convert native mappings into R2RML mappings and vice versa, and the R2RML version of the mappings introduced here is available in the tutorial GitHub repository.

**Figure 3:**
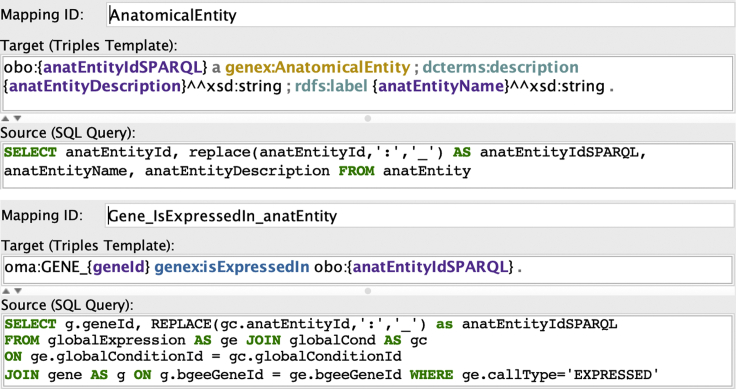
Examples of *Ontop* mapping assertions from the EasyBgee relational DB to a KG based on gene expression ontology

To illustrate the use of mappings, let us consider the mapping assertion depicted in the upper part of Figure 3, between the EasyBgee DB and the GenEx ontology:

1. The mapping identifier is AnatomicalEntity.
2. The source part is an SQL query over the relation anatEntity, with answer variables anatEntityId, anatEntityIdSPARQL, anatEntityName, and anatEntityDescription (see also the data schema in Figure 2).
3. The target part consists of the following three RDF triple patterns, which refer to the answer variables of the SQL query (shown in violet, in Figure 3).

Notice that in Figure 3 we have used the standard abbreviated notation for RDF triple patterns (and triples), where one avoids to repeat the subject in multiple triples with the same subject, by separating these triples with ‘; ’ (instead of ‘.’).

When designing the mapping, we can take advantage of SQL functions and existing ontologies to deal with potential semantic and data heterogeneity in the source data. As illustrated in Figure 3, we use the SQL function replace() to modify the anatomical entity identifiers stored in the anatEntity table by replacing each ‘:’ with ‘_.’ In addition, we prepend the obo: prefix to the modified ids in order to obtain the exact corresponding UBERON term, such as the obo:UBERON_0000955 term labeled as *brain*. Another example is a mapping assertion for the species table, where we use the SQL function concat() to concatenate the values from the two columns species.genus and species.species into a single value, which is then assigned as a value for the property up:scientificName of a species from the UniProt core ontology,^39^ which GenEx imports.

**Figure 4:**
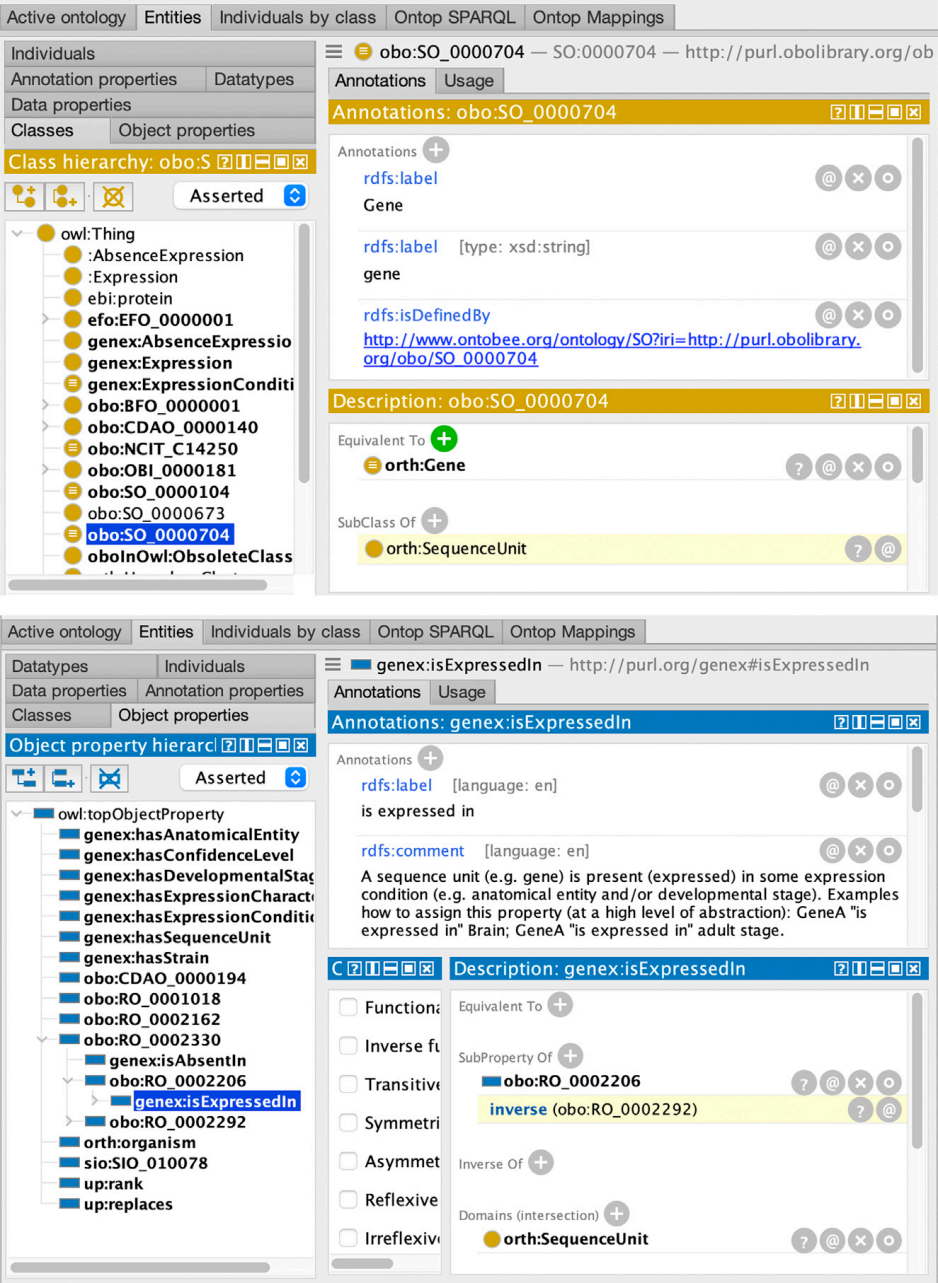
Class view and property view of Protégé The top part of the figure shows the *class view* of Protégé. The selected class obo:SO_0000704 has been declared to be as equivalent to the class orth:Gene. The bottom part of the figure shows the *property view* of Protégé. The selected property genex:isExpressedIn has been declared as a sub-property of obo:RO_0002206 and inferred as a sub-property of the inverse of the obo:R0_0002292 property. This inference occurs because obo:RO_0002206 is explicitly defined as the inverse of obo:R0_0002292.

A relevant feature that we can exploit when designing ontology and mapping in the VKG approach is the possibility to semantically enrich the original data sources by making implicit information explicit. To illustrate such semantic enrichment, let us consider the second mapping assertion in Figure 3. There is no foreign key constraint in the original DB directly relating a gene (in the gene table) to an anatomical entity (in the anatentity table), as shown in Figure 2. Moreover, there is no column or table stating the explicit relation between genes and anatomic entities, as the one defined in the GenEx ontology by means of the genex:isExpressedIn property. Nonetheless, by means of the mapping assertion with id Gene_IsExpressedIn_anatEntity, the VKG system is able to assert genex:isExpressedIn properties (between instances of the classes orth:Gene and genex:AnatomicalEntity). In addition, thanks to the reasoning capabilities of *Ontop* and the sub-property and inverse property axioms in the GenEx ontology discussed in the section “The gene expression ontology” (see also bottom part of Figure 4), the system automatically derives also data assertions for the property obo:RO_0002292 (i.e., “expresses”). This is because genex:isExpressedIn is a sub-property of the inverse of obo:RO_0002292. Therefore, although we explicitly specified only a mapping assertion for the genex:isExpressedIn property, when the VKG system retrieves the corresponding data assertions, due to its reasoning capabilities, it infers also data assertions for the property obo:RO_0002292.

Similarly to inferences concerning the RO property “expresses,” via reasoning the VKG system is also capable of inferring instances of equivalent classes, i.e., classes that have been aligned in the ontology by means of owl:equivalentClass^36^ statements. For example, GenEx states that orth:Gene from the othology ontology (ORTH)^40^ and obo:SO_0000704 (which stands for “gene”) from the sequence ontology (SO) are equivalent, as shown in the top part of Figure 4. Therefore, the instances of one concept are also instances of the other one and vice versa, although in the VKG specifications we include only a mapping assertion for the orth:Gene concept. As a result, we can interchangeably use orth:Gene and obo:SO_0000704, and consider them as synonyms (see query 2 in “Querying the KG”).

### Designing the ontology and mapping

As we have seen in the previous section, to enable the VKG approach one needs an ontology describing the domain of interest and a mapping populating such ontology starting from the content of the database.

Designing an ontology is not an easy task, and in many domains (e.g., the biomedical one) ontologies are developed independently by trained experts and are already available to be re-used. However, if a domain ontology that suits the user requirements is not available, the user can rely on the open-source tool Protégé shown in this tutorial, which provides a graphical interface that helps in designing an ontology from scratch or in modifying an already existing one. Protégé also comes with a set of plugins for debugging and visualizing the ontology, and integrated reasoners that help the conceptual modeling activity.

Writing a mapping manually is a time-consuming, error-prone task, and automatic approaches to mapping generation are still an open research topic. Notable recent developments in this area involve both foundational research^41^^,^^42^ and implemented systems.^41^^,^^43^

## Querying the KG

We are now ready to query the KG, created as described in the previous sections, through the SPARQL query language, a W3C recommendation for querying KGs.^19^ We rely on the *Ontop* plugin for the ontology editor Protégé,^44^ which provides a graphical user interface both for managing the mapping between a DB and an ontology, and also for specifying and executing queries over the resulting KG specification in the virtual setting. The *Ontop* plugin visualizes the result of the query in a dedicated window.

We illustrate the main ideas behind query answering over a KG on three example queries.

### Query 1

**Figure 5:**
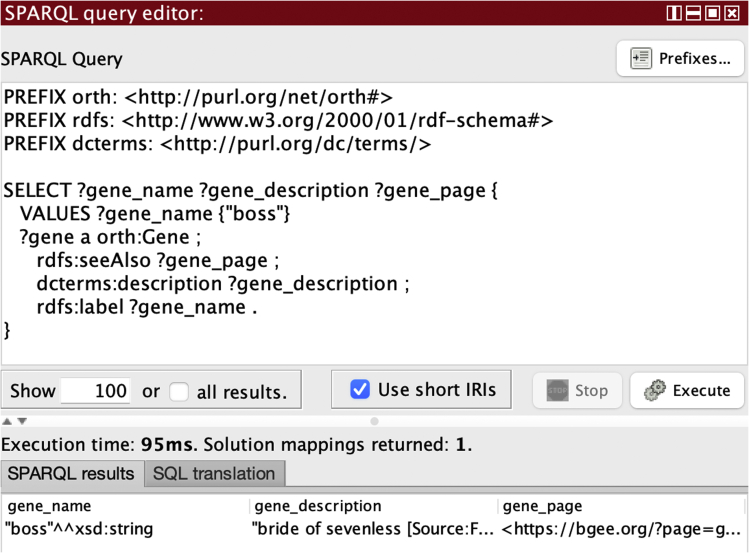
SPARQL query interface of the *Ontop* plugin for Protégé Query 1 and the result of its execution are shown.

The query $Q_{1}$ shown in Figure 5 asks for the gene information that is associated to a given gene name, in this case the “boss” (the bride of sevenless) gene product that acts as a ligand for the sevenless tyrosine-kinase receptor during eye development.^45^ Figure 5 shows the graphical interface provided by the *Ontop* plugin for Protégé for formulating SPARQL queries and visualizing the result of their execution. The bottom part of the window shows the result of the execution of $Q_{1}$, which in case is a single set of bindings for the three answer variables of the SPARQL query.

### Query 2

The following query $Q_{2}$ retrieves the anatomical entities, such as organs, where the gene labeled "boss" is expressed. We observe that results are still returned, although $Q_{2}$ asks for individuals of the non-mapped class obo:SO_0000704. This happens since the ontology states that obo:SO_0000704 (which is labeled as gene) from SO is equivalent to the class orth:Gene from ORTH, and reasoning over the ontology axioms is applied. Intuitively, this happens by *rewriting* the query into one that uses the term orth:Gene, instead of obo:SO_0000704, and by retrieving results also for this rewritten query. Therefore, we can interchangeably use both terms, although we only wrote a mapping assertion for orth:Gene. As already mentioned in the previous section, this simplifies the design of the mapping, because it allows one to avoid to extensively and explicitly write a mapping assertion for each term in the ontology.

SELECT DISTINCT ?organ {

VALUES ?gene_name {"boss"}

?gene a obo:SO_0000704 ;

# equivalent to orth:Gene

rdfs:label ?gene_name ;

genex:isExpressedIn ?organ .

?organ a genex:AnatomicalEntity .

}

### Query 3

Consider now the following query $Q_{3}$, which retrieves all genes expressed in the brain.

SELECT  ?gene_name ?gene_page {

?organ  obo:RO_0002292 ?gene ;

rdfs:label "brain" .

?gene  rdfs:seeAlso ?gene_page ;

rdfs:label ?gene_name .

}

By reasoning over the axioms that state sub-properties and inverse properties in the ontology, we can safely expect to get results for $Q_{3}$, although we did not introduce in the VKG system any specific mapping assertion for the property obo:RO_0002292 used in the query. In this context, we exploit the axioms stating that obo:RO_0002292 is the inverse property of obo:RO_0002206, and that genex:isExpressedIn from Genex is a sub-property of the latter. Similarly to what happened for $Q_{2}$, the query-rewriting algorithm enriches $Q_{3}$ with a query making use of genex:isExpressedIn instead of obo:RO_0002292, and since we introduced in the VKG system a mapping assertion for genex:isExpressedIn, we can obtain indeed answers using the data stored in the underlying DB.

## Deployment

Once the VKG has been developed, we can also make it available to external users. To do so, we can follow two approaches: (1) materialize the RDF triples into a file, which can then be uploaded to a file server and downloaded by other users; (2) set up an SPARQL endpoint so that users can query it. We now discuss these two options.

### Materialization

For VKGs over a dataset of small size, one can use the *Ontop* plugin for Protégé to materialize the triples that make up the KG. Otherwise, it is recommended to use the command line interface (CLI) of *Ontop*. To do so, one can download *Ontop* CLI, unzip it, and invoke *Ontop* passing it the “materialize” directive. For example, to use the files provided in the online GitHub repository accompanying this tutorial, one can issue the following command (the --ontology and --mapping options are used to specify the files containing, respectively, an OWL 2 QL ontology and a set of mapping assertions (in the specific *Ontop* syntax), while the file supplied with the --properties option contains the connection parameter for the DB):

ontop materialize \

--ontology=bgee_v14_genex.owl \

--mapping=bgee_v14_genex.obda \

--properties=bgee_v14_genex.properties \

--output=bgee_v14_genex.ttl

Then the triples are materialized into the file bgee_v14_genex.ttl. Such a file can be shared and further analyzed, or it can be loaded into a triple store.

### SPARQL endpoint

Setting up a SPARQL endpoint makes the VKG queryable as a standard HTTP service. This can be done either through a manual setup using the CLI of *Ontop*, or through a container-based deployment using Docker. We discuss now both options.

#### Using the CLI on *Ontop*

The following command starts the SPARQL endpoint and makes it available at URL http://localhost:8080/sparql.

ontop endpoint \

--ontology=bgee_v14_genex.owl \

--mapping=bgee_v14_genex.obda \

--properties=bgee_v14_genex.properties \

--portal=bgee_v14_genex.toml

The SPARQL endpoint may be accessed using any HTTP client, including SPARQL clients and tools using the standard SPARQL HTTP protocol. For instance, using curl^46^:

curl --request POST \

--url http://localhost:8880/sparql \

--header 'accept: application/json' \

--header \

'content-type: application/sparql-query' \

--data 'SELECT * { ?s ?p ?o } LIMIT 5’

The endpoint also comes with a handy web interface at http://localhost:8080, where users can formulate SPARQL queries.

#### Using the *Ontop* Docker image

We have also developed a Docker-based deployment (defined in the docker-compose.yml file), which consists of two services: one for the MySQL DB, and another for the *Ontop* SPARQL endpoint. With this setup, one avoids manually configuring the MySQL DB and installing Java and *Ontop*. Instead, the following single command starts the whole tutorial, with the same services as those offered by the CLI:

docker-compose up

## Conclusions

In this tutorial we have learned how to set up and exploit the VKG approach to access data stored in relational legacy systems, and to enrich such data with domain knowledge coming from different heterogeneous (biomedical) resources. Specifically, we have shown how the gene expression ontology can be mapped to the EasyBgee database to expose its content as a KG, and how to query such a KG by means of the VKG system *Ontop*. All the software artifacts presented in this tutorial (ontology, mappings, data, etc.) are available through the online repository.^22^
